# Acute Corrosive Injuries of the Stomach: A Single Unit Experience of Thirty Years

**DOI:** 10.5402/2011/914013

**Published:** 2010-10-28

**Authors:** N. Ananthakrishnan, G. Parthasarathy, Vikram Kate

**Affiliations:** Department of Surgery, Jawaharlal Institute of Postgraduate Medical Education and Research, Pondicherry 605006, India

## Abstract

*Introduction*. The spectrum of gastric injury due to corrosives can vary. This paper presents a single center experience of over 30 years of corrosive gastric injuries of 39 patients with acute gastric injuries from 1977 till 2006. *Patients and Methods*. Two thirds of the patients in the acute injury group had a concomitant esophageal injury. The age of the patients ranged from 4 years to 65 years with a slight preponderance of males. (M : F ratio 22 : 17). *Results*. 36 out of 39 acute gastric injuries were due to ingestion of acids. Three patients had history of caustic soda ingestion. Oral hyperemia or ulcers of varying extent were seen in all patients. The stomach showed hyperemia in 10, extensive ulcers in 13, and mucosal necrosis in 10 patients. Fifteen patients (15/39, 38.5%) were managed conservatively. Twenty four patients (24/39, 61.5%) underwent laparotomy: one for frank peritonitis, 10 for gastric mucosal necrosis, and 13 others for extensive gastric ulcerations. Overall the mortality rate was 29.6 %. *Conclusion*. Although the mortality and morbidity of acute corrosive gastric injuries is high, the key to improve the survival is early identification of perforation, maintenance of nutrition and control of sepsis.

## 1. Introduction

Corrosive injuries of the stomach are not uncommon in developing countries. In these countries, accidental or suicidal ingestion of acids is encountered more often than in developed countries where lye or alkaline corrosives are more frequent [1]. Both accidental ingestion, particularly in children, due to careless storing of chemicals and ingestion with suicidal intent and due to free availability of the caustic agents contribute to their occurrence. 

The relative extent of esophageal and gastric involvement largely depends on the nature of the corrosive ingested. Acids affect the stomach more commonly than alkalis [2], cause mucosal damage by coagulation necrosis, and require a longer duration of contact [3]. However, alkali damage of the stomach has also been reported [4, 5]. Acids are cleared rapidly from the esophagus to the stomach where they pool in the prepyloric area due to corrosive-induced pylorospasm [6–8]. Prolonged contact with the prepyloric mucosa results in a prepyloric stricture. Strictures can also occur in the antrum, body, or in the pyloroduodenal area. When the volume of the corrosive ingested is large, the entire stomach gets scarred leading to a diffusely contracted stomach. On the other hand, alkalis cause liquefaction necrosis [3], are more viscous, and tend to adhere to the esophageal mucosa with only a relatively small amount reaching the stomach. The extent of esophageal damage is greater with alkalis than acids. 

Extensive acute injuries are usually fatal and therefore, the spectrum of acute and chronic gastric injury seen at a tertiary care referral hospital is not reflective of the overall picture as patients with the most severe gastric and esophageal injuries die at peripheral centers. 

The spectrum of gastric injury due to corrosives can vary from acute partial or total gastric mucosal or transmural necrosis to chronic gastric injuries of different types. This article presents a single center experience of over 30 years of corrosive gastric injuries of 39 patients with acute gastric injuries emphasizing the spectrum of injuries and the extent of involvement and highlighting the possible modes of management.

## 2. Patients and Methods

Thirty nine consecutive patients with injury to the stomach following ingestion of a corrosive were treated in our institute from 1977 till 2006. Two thirds of the patients in the acute injury group had a concomitant esophageal injury. The age of the patients ranged from 4 years to 65 years with a slight preponderance of males over females (male : female ratio of 22 : 17).

## 3. Results

There were a total of 39 acute and 109 chronic gastric injuries seen at our institute. The ratio 39 : 109 (1 : 2.8) between acute and chronic injuries does not reflect the true proportion between acute and chronic injuries in real life as several patients with acute injuries die at peripheral hospitals without reaching a tertiary care centre. Also a number of chronic gastric injuries were referred to us because of the interest of the unit in this problem.

36 out of 39 acute gastric injuries were due to ingestion of acids (Aqua regia : 15 and bathroom cleaning acid : 21).These are readily available: the former being used by goldsmiths and the latter being freely available commercially. Three patients had history of caustic soda ingestion (Table 1).

There were 6 children less than 12 years of age, all of whom had ingested the corrosive accidentally. Of the 33 adults, 21 were suicidal and 12 accidental ingestions. All patients with acute corrosive injury presented in a critical condition with abdominal pain, vomiting, and hematemesis. These patients also had severe odynophagia and 24/39 (61.5%) in addition, had dysphagia and drooling of saliva. One patient with previous history of a truncal vagotomy and gastrojejunostomy for benign gastric outlet obstruction due to chronic cicatrizing duodenal ulcer presented with features of peritonitis following perforation of the efferent limb of the gastrojejunostomy. None of the remaining patients had frank features of esophageal or abdominal hollow viscus perforation.

**Figure 1:**
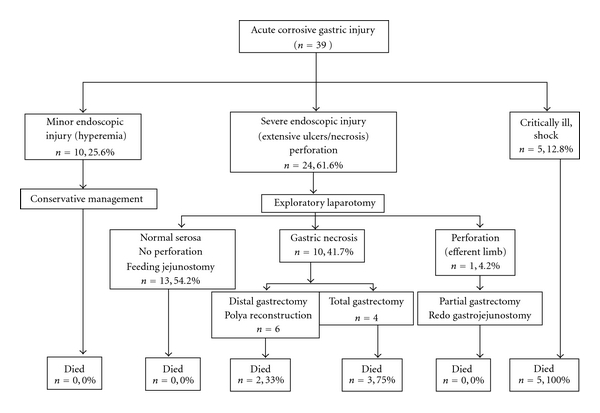
Flow chart showing details of management of acute corrosive gastric injuries.

The details of endoscopic appearance and clinical course are shown in Figure 1. Endoscopic evaluation was performed in 33/39 patients. One patient with overt peritonitis and five patients who were critically ill and in shock did not undergo endoscopic evaluation. The timing of endoscopy varied from patient to patient depending on the clinical condition. However, where feasible it was the policy of the unit to do endoscopy 3-4 days after the injury to have a true picture of the extent of the damage. Endoscopy was carefully performed with minimal air insufflation. 

Oral hyperemia or ulcers of varying extent were seen in all patients. Twenty four patients had grade 2 or 3 esophageal injury. The stomach showed hyperemia in 10, extensive ulcers in 13, and varying degrees of mucosal necrosis in the remaining 10 patients. 

A total of fifteen patients (15/39, 38.5%) were managed conservatively. Of these, five patients were critically ill, while 10 patients had only minor endoscopic evidence of injury (hyperemia). The conservative management included nil per oral status (NPO), placement of an indwelling nasogastric tube, and starting of parenteral feeding. One patient, who had extensive laryngeal burns and stridor, needed an emergency tracheostomy for airway control. The five patients who presented in shock died within 24 hours. In the remaining ten patients, tube feeds were resumed successfully after one week gradually progressing to oral feeds over the next few days. 

Twenty four patients (24/39, 61.5%) underwent exploratory laparotomy: one for frank peritonitis, 10 with gastric mucosal necrosis on endoscopy, and 13 others with extensive gastric ulcerations considered at high risk for perforation. In 13 patients, the stomach appeared relatively normal on the serosal aspect at laparotomy with no evidence of perforation. Feeding jejunostomy were the sole procedure carried out in these patients. In one patient, there were perforation of the efferent loop of a previous gastrojejunostomy. Partial gastrectomy and redo gastrojejunostomy was done. In 10 patients, there was varying degrees of gastric necrosis. The extent of mucosal necrosis, in general, was much more as seen at gastrotomy as compared to the extent of transmural necrosis seen from the serosal side. In six patients, a distal gastrectomy and a Polya reconstruction (end to side gastrojejunostomy) were performed along with a feeding jejunostomy. In the other four, a total gastrectomy was carried out. Due to the critical condition of the patients, no reconstruction was attempted. A cervical esophagostomy was done. The abdominal esophagus was closed around a drainage tube, the duodenal stump was closed with drainage, and a feeding jejunostomy was done. The details of the management of patients with acute corrosive injuries of the stomach are shown in Figure 1.

Overall, the mortality rate was 29.6% (Figure 1). All patients who presented in shock, 3 of 4 with total gastrectomy and 2 of 6 with distal gastrectomy, expired due to sepsis and shock.

## 4. Discussion

Corrosive injuries of the stomach and esophagus are not infrequent causes of hospitalization in countries like India [1]. Both accidental ingestion, particularly in children, due to careless storing of chemicals and ingestion with suicidal intent due to free availability of the caustic agents contribute to their occurrence. In most reported series, the chemicals that are most commonly responsible are alkalis like potassium and sodium hydroxide [7, 9–11]. In contrast, the majority of the corrosive injuries in India are due to acids. The most common acids implicated are bathroom cleaning acid (concentrated hydrochloric acid) and aqua regia.

Two thirds of the patients with acute gastric injury in the present series had a concomitant esophageal injury. The incidence of coexistent esophageal injury in the literature varies from 20% to as high as 62.5% [12–14].

The most common presentation of an acute corrosive gastric burn is with abdominal pain, vomiting, and hematemesis [12, 15, 16]. Rarely a full thickness burn can cause a gastric perforation. This normally tends to present a few days after ingestion of the corrosive. Hematemesis following corrosive ingestion is usually self-limiting. However, there are reports of subacute massive bleeding from the stomach or duodenum following corrosive ingestion [17]. Massive bleeding typically occurs two weeks after ingestion. Such bleeds may warrant a gastrectomy if the source is the stomach or a duodenotomy and under running of the bleeding vessel in the duodenum. 

The most useful investigation in the evaluation of an acute corrosive gastric injury is an upper gastrointestinal endoscopy. Endoscopic evaluation has been advised as soon as possible after corrosive ingestion, since it is believed that the risk of perforation is lowest at this point [3]. Also, it is believed that endoscopic evaluation at this juncture helps plan early intervention, if required. However, in our experience clinical features and radiological examination in the form of a CT are more useful in assessing threatened or existing perforation. Early endoscopy carries the risk of misdiagnosing the extent of transmural damage in the presence of extensive hyperemia. A repeat study is again required a few days subsequently to assess the true damage. It is our policy therefore, to do endoscopy 72–96 hours after the corrosive ingestion.

 Laparoscopy is also a useful adjunct in assessing a patient who has a high risk of gastric perforation as seen on endoscopy or in patients with severe esophageal injury in whom an upper gastrointestinal endoscopy to assess the stomach is not feasible. Some authors have advocated routine laparoscopic examination in all injuries of second degree or greater [18]. However, this has not been our practice. There is also a report on the use of a Meckel's scan to assess the severity of the gastric injury [19].

Following an acute injury to the stomach by corrosive ingestion, the initial management is usually conservative. Adherence to the basic tenet of avoiding a gastric lavage in any corrosive poisoning cannot be overemphasized. The patient usually has associated burns to the upper aerodigestive tract which needs attention as well, if required, with a tracheostomy. Attempts at neutralizing the acids or alkalis are ill-advised and the resulting exothermic reaction from the neutralization process may do more harm than good [20]. Similarly, there is not much role for measures to dilute the corrosive with milk, water and so forth, as the definitive extent of the injury is determined within minutes after ingestion [18]. 

Emergency surgical intervention is needed if the patient develops any signs of esophageal perforation, peritonitis, or uncontrolled massive hematemesis [14]. In view of the high probability of slow but relentless progression of transmural necrosis, there should be a low threshold for consideration of laparotomy at the earliest suspicion. If there is severe esophageal burn with a high likelihood of stricture formation, a feeding jejunostomy is performed and the stomach is assessed intraoperatively at this time. If the esophagus is relatively spared with moderate injury to the stomach, the patient is fed through a jejunostomy and kept on regular observation to monitor the progress of the gastric burn. If, on the other hand, the stomach appears soft and necrotic, a gastrotomy is made and the extent of transmural and mucosal necrosis is assessed before planning a resection, either a distal gastrectomy or a total gastrectomy.

Patients with extensive gastric injury are often critically ill and do not withstand lengthy reconstructive procedures. Hence, as a policy for extensive gastric necrosis, we do total gastrectomy, closure and drainage of the esophageal, and duodenal stump, a cervical esophagostomy along with a feeding jejunostomy, leaving reconstruction (using a jejunal loop) for a more opportune moment should the patient comes out of the acute phase. For less extensive acute gastric injury a distal gastrectomy may suffice. The line of section should be decided after gastrotomy since mucosal necrosis is more extensive than what is apparent from the serosal side. There is no role for procedures such as closure of a perforation since the stomach is like wet blotting paper around the site of perforation.

All patients with second degree or greater corrosive burns are given parenteral broad spectrum antibiotics. Intravenous proton pump inhibitors are also widely used with the aim of minimizing the insult to the injured gastric mucosa. However, there are no studies supporting their role in this setting. 

The debate over the use of steroids in corrosive burns to prevent stricture formation has been put to rest with two recent meta-analyses [21, 22].The authors found no benefit with the use of systemic corticosteroids in corrosive ingestion and proscribe their routine use to prevent stricture formation. However, there is a report of the use of intralesional steroids in corrosive pyloric strictures [23].

The mandatory need for gastric resection as prophylaxis against future malignancy has been overstated in the literature. There have been reports of malignancy developing in a scarred esophagus or stomach following corrosive ingestion [24–26]. However, in our experience this association has been found to be tenuous. In an experience of over 500 corrosive injuries seen over a thirty year period, there was only one solitary instance of cricopharyngeal carcinoma following esophageal burns by caustic ingestion and one case of peri-gastroenterostomy carcinoma seventeen years after the ingestion of acid. In the latter, it is not clear whether the carcinoma was corrosive induced or secondary to chronic bile reflux through the gastrojejunostomy stoma (stump carcinoma or postgastric surgery carcinoma).

The mortality and morbidity of acute corrosive gastric injuries are high and dependent on the severity of initial damage caused by the corrosive agent with a significant proportion of patients succumbing to their injuries either before reaching tertiary care or soon thereafter. The key to improving the survival of such patients in the acute setting remains in early identification of perforation and supportive care with maintenance of nutrition and control of sepsis. One has to be pithy in one's surgical interventions for acute corrosive burns, limiting the resection to only the grossly injured bowel and leaving the reconstruction part for a latter day. On the other hand, the mortality and morbidity of chronic gastric corrosive injuries can be significantly reduced by adequate preoperative preparation and a planned protocol of approach dependent on the type of injury.

## Figures and Tables

**Table 1:** Nature of corrosive ingested.

Acute Gastric Injury (*n* = 39)	
Acids	36
Aqua regia*	15
Bathroom cleaning acid^+^	21
Alkalis	3

*A mixture of hydrochloric and nitric acids used by goldsmiths as a solvent,

^+^Concentrated nitric acid
